# Supplementary material for: Oral supplementation of gut microbial metabolite indole-3-acetate alleviates diet-induced steatosis and inflammation in mice
Source: eLife. 2024 Feb 27;12:RP87458. doi: 10.7554/eLife.87458 (PMC10942630; doi:10.7554/eLife.87458)
Supplement: Figure 6—source data 2. [file elife-87458-fig6-data2.pptx]

## Slide 1
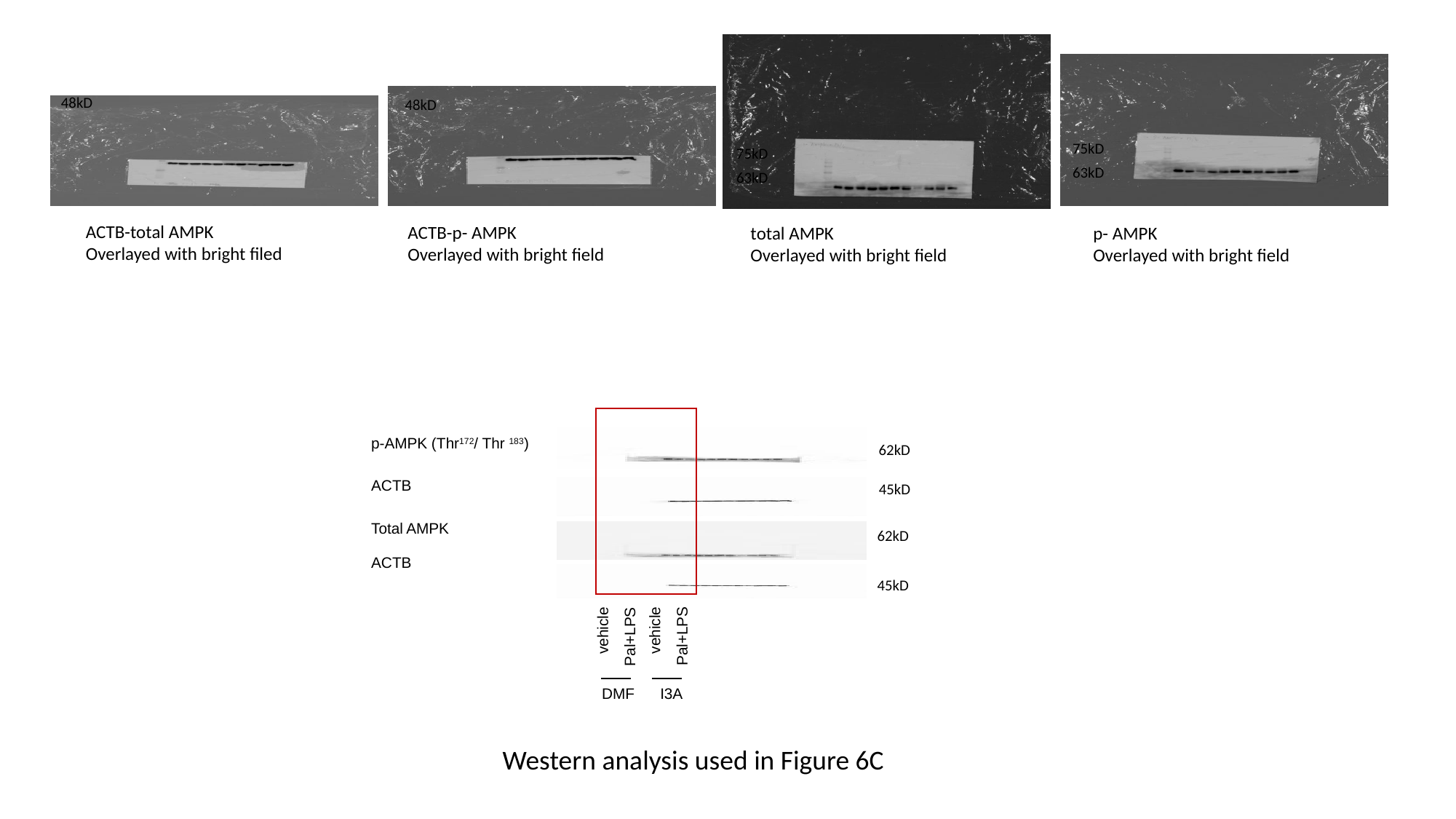

48kD
48kD
75kD
75kD
63kD
63kD
ACTB-total AMPK
Overlayed with bright filed
ACTB-p- AMPK
Overlayed with bright field
p- AMPK
Overlayed with bright field
total AMPK
Overlayed with bright field
p-AMPK (Thr172/ Thr 183)
62kD
ACTB
45kD
Total AMPK
62kD
ACTB
45kD
vehicle
Pal+LPS
vehicle
Pal+LPS
I3A
DMF
Western analysis used in Figure 6C
