# Supplementary material for: Oral supplementation of gut microbial metabolite indole-3-acetate alleviates diet-induced steatosis and inflammation in mice
Source: eLife. 2024 Feb 27;12:RP87458. doi: 10.7554/eLife.87458 (PMC10942630; doi:10.7554/eLife.87458)
Supplement: Figure 6—figure supplement 1—source data 1. [file elife-87458-fig6-figsupp1-data1.pptx]

## Slide 1
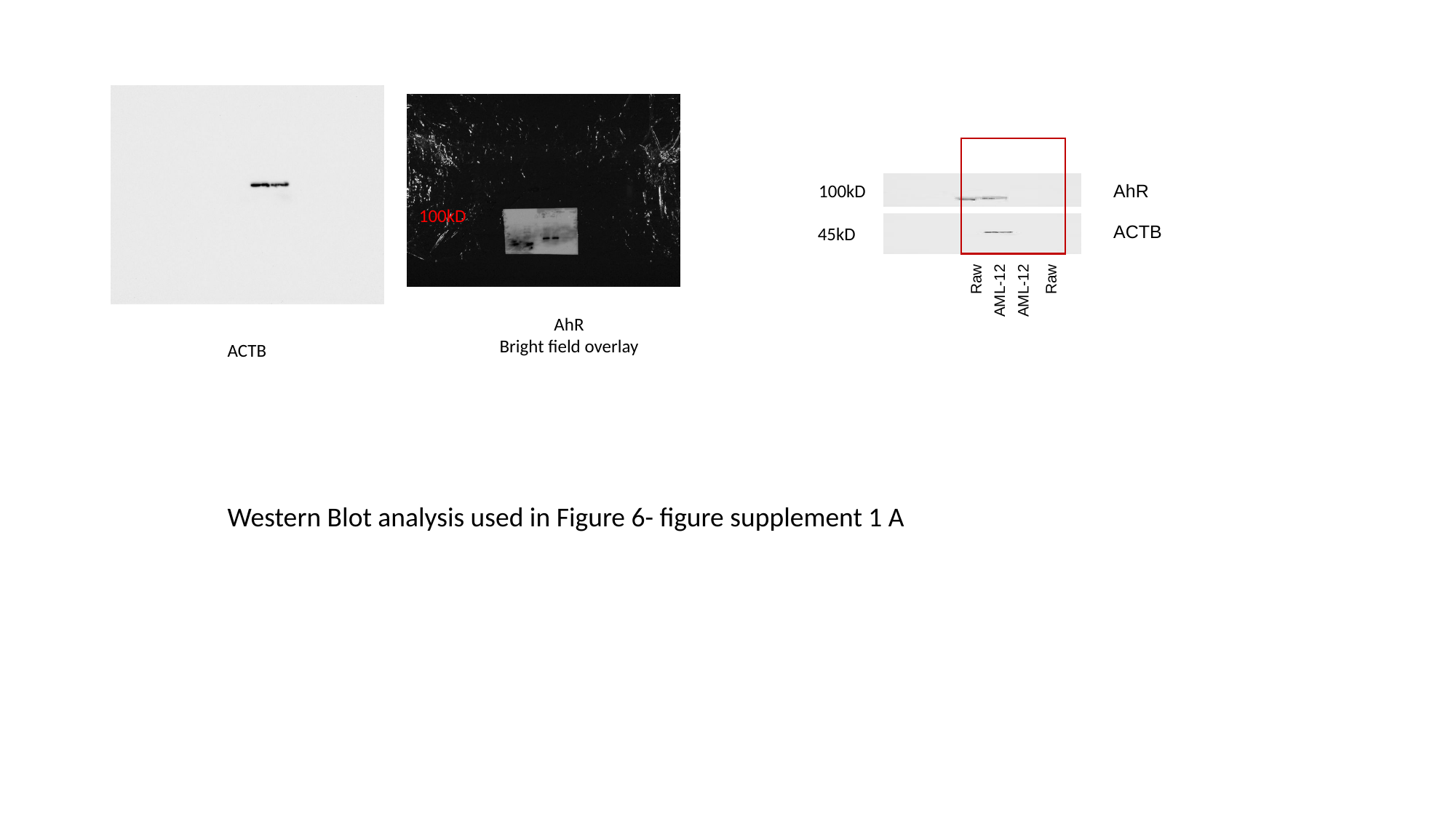

100kD
AhR
100kD
ACTB
45kD
Raw
AML-12
AML-12
Raw
AhR
Bright field overlay
ACTB
Western Blot analysis used in Figure 6- figure supplement 1 A
