# Supplementary material for: Oral supplementation of gut microbial metabolite indole-3-acetate alleviates diet-induced steatosis and inflammation in mice
Source: eLife. 2024 Feb 27;12:RP87458. doi: 10.7554/eLife.87458 (PMC10942630; doi:10.7554/eLife.87458)
Supplement: Figure 6—figure supplement 2—source data 1. [file elife-87458-fig6-figsupp2-data1.pptx]

## Slide 1
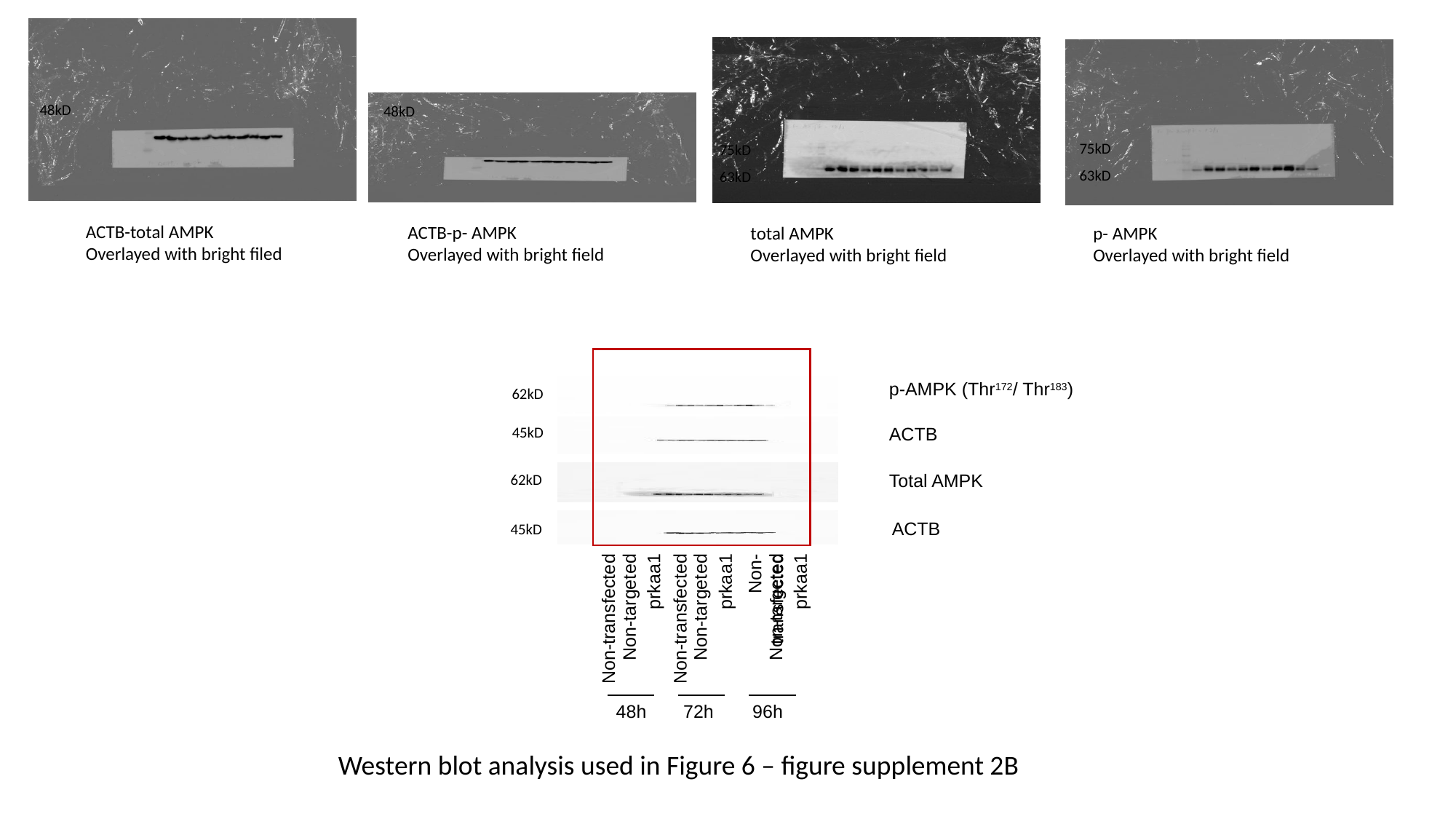

48kD
48kD
75kD
75kD
63kD
63kD
ACTB-total AMPK
Overlayed with bright filed
ACTB-p- AMPK
Overlayed with bright field
p- AMPK
Overlayed with bright field
total AMPK
Overlayed with bright field
p-AMPK (Thr172/ Thr183)
62kD
ACTB
45kD
Total AMPK
62kD
ACTB
45kD
prkaa1
Non-targeted
Non-targeted
Non-transfected
prkaa1
prkaa1
Non-targeted
Non-transfected
Non-transfected
48h
72h
96h
Western blot analysis used in Figure 6 – figure supplement 2B
