# Supplementary material for: Oral supplementation of gut microbial metabolite indole-3-acetate alleviates diet-induced steatosis and inflammation in mice
Source: eLife. 2024 Feb 27;12:RP87458. doi: 10.7554/eLife.87458 (PMC10942630; doi:10.7554/eLife.87458)
Supplement: Supplementary file 1. — Solvent A was formic acid solution in water (0.1% vol/vol). Solvent B was a 0.1% vol/vol formic acid solution in methanol. The flow rate used was 0.4 ml/min. [file elife-87458-supp1.docx]

Supplementary File 1. Chromatography gradient method for untargeted metabolomics

| **Time (min)** | **% Solvent A** | **% Solvent B** |
| --- | --- | --- |
| 0 | 90 | 10 |
| 5 | 60 | 40 |
| 7 | 5 | 95 |
| 9 | 5 | 95 |
| 9.1 | 90 | 10 |
| 13 | 90 | 10 |

Solvent A: 0.1% (v/v) formic acid solution in water. Solvent B was a 0.1% (v/v) formic acid solution in methanol. The flow rate was 0.4 mL/min.
