# Supplementary material for: Oral supplementation of gut microbial metabolite indole-3-acetate alleviates diet-induced steatosis and inflammation in mice
Source: eLife. 2024 Feb 27;12:RP87458. doi: 10.7554/eLife.87458 (PMC10942630; doi:10.7554/eLife.87458)
Supplement: Supplementary file 2. — For the chromatographic method, solvent A was ammonium acetate (2 mM) in water and solvent B was methanol:acetonitrile (50:50, vol/vol). The flow rate used was 0.4 ml/min. [file elife-87458-supp2.docx]

Supplementary File 2. Chromatography gradient and LC-MS parameters for bile acid analysis

| **Time (min)** | **% Solvent A** | **% Solvent B** |
| --- | --- | --- |
| 0 | 55 | 45 |
| 9 | 30 | 70 |
| 9.5 | 30 | 70 |
| 9.51 | 55 | 45 |
| 12 | 60 | 40 |

| **Bile acid** | **RT**  **(min)** | **Polarity** | **Precursor**  **(m/z)** | **Product**  **(m/z)** | **CE**  **(V)** | **RF Lens**  **(V)** |
| --- | --- | --- | --- | --- | --- | --- |
| Muricholic acid | 1.39 | Negative | 407.3 | 371.3 | 31.7 | 249 |
| Tauromuricholic acid | 0.9 | Negative | 514.5 | 79.9 | 55 | 185 |
| Tauro-β-muricholic acid | 1.87 | Negative | 514.5 | 80 | 55 | 248 |
| Taurohyodeoxycholic acid | 1.58 | Negative | 498.6 | 79.9 | 55 | 137 |
| Murideoxycholic acid | 2.54 | Negative | 391.5 | 327.3 | 34.3 | 170 |
| Glycocholic acid | 1.76 | Negative | 464.5 | 74 | 36.8 | 111 |
| Taurocholic acid | 1.87 | Negative | 514.5 | 79.9 | 55 | 137 |
| Ursodeoxycholic acid | 2.2 | Negative | 391.4 | 355.2 | 32.4 | 178 |
| Cholic acid | 2.64 | Negative | 407.8 | 290.3 | 37.5 | 131 |
| Hyodeoxycholic acid | 2.54 | Negative | 391.5 | 353.2 | 35.7 | 173 |
| Taurodeoxycholic acid | 3.05 | Negative | 498.4 | 79.9 | 54.6 | 198 |
| Deoxycholic acid | 4.83 | Negative | 391.4 | 327.2 | 34.6 | 146 |
| Chenodeoxycholic acid | 4.6 | Negative | 391.4 | 355.1 | 34.8 | 154 |
