# Supplementary material for: Oral supplementation of gut microbial metabolite indole-3-acetate alleviates diet-induced steatosis and inflammation in mice
Source: eLife. 2024 Feb 27;12:RP87458. doi: 10.7554/eLife.87458 (PMC10942630; doi:10.7554/eLife.87458)
Supplement: Supplementary file 3. — For the chromatographic method, solvent A was acetonitrile/water (3:2, vol/vol) containing 10 mM ammonium acetate. Solvent B was acetonitrile/isopropanol (1:1, vol/vol). The injection volume was 5 μl and the oven temperature was set to 55°C. [file elife-87458-supp3.docx]

Supplementary File 3. Chromatography gradient and LC-MS parameters for free fatty acid analysis

| **Time (min)** | **% Solvent A** | **% Solvent B** |
| --- | --- | --- |
| 0 | 90 | 10 |
| 1.7 | 90 | 10 |
| 11.9 | 65 | 35 |
| 14.9 | 0 | 100 |
| 17.4 | 0 | 100 |
| 17.9 | 90 | 10 |
| 20 | 90 | 10 |

| **Free fatty acids** | **RT (min)** | **[M-H]- (m/z)** | **TOF MS^2^ (m/z)** | **CE (V)** | **DE** |
| --- | --- | --- | --- | --- | --- |
| Light Experiment for quantification | | | | | |
| Lauric acid | 1.94 | 199.17 | 199.17 | -8 | -100 |
| Myristic acid | 3.28 | 227.2 | 227.2 | -15 | -150 |
| Palmitic acid | 6.02 | 255.23 | 255.23 | -15 | -150 |
| Palmitoleic acid | 3.91 | 253.22 | 253.22 | -15 | -150 |
| Stearic acid | 10.81 | 283.26 | 283.26 | -15 | -150 |
| Oleic acid | 7.01 | 281.25 | 281.25 | -15 | -150 |
| Linoleic acid | 4.79 | 279.22 | 279.22 | -17 | -150 |
| Linolenic acid | 3.38 | 277.22 | 277.22 | -15 | -150 |
| Arachidonic acid | 4.57 | 303.23 | 303.23 | -10 | -175 |
| Heavy Experiment for confirmation | | | | | |
| Lauric acid | 1.94 | 199.17 | 199.17 | -21 | -100 |
| Myristic acid | 3.28 | 227.20 | 227.20 | -23 | -150 |
| Palmitic acid | 6.02 | 255.23 | 255.23 | -26 | -150 |
| Palmitoleic acid | 3.91 | 253.22 | 253.22 | -25 | -150 |
| Stearic acid | 10.81 | 283.26 | 283.26 | -25 | -150 |
| Oleic acid | 7.01 | 281.25 | 281.25 | -26 | -150 |
| Linoleic acid | 4.79 | 279.22 | 279.22 | -25 | -150 |
| Linolenic acid | 3.38 | 277.22 | 277.22 | -23 | -150 |
| Arachidonic acid | 4.57 | 303.23 | 303.23 | -20 | -175 |
