# Supplementary material for: Oral supplementation of gut microbial metabolite indole-3-acetate alleviates diet-induced steatosis and inflammation in mice
Source: eLife. 2024 Feb 27;12:RP87458. doi: 10.7554/eLife.87458 (PMC10942630; doi:10.7554/eLife.87458)
Supplement: Supplementary file 4. [file elife-87458-supp4.docx]

Supplementary File 4. Chromatography gradient method for untargeted proteomics

| **Time (min)** | **% Solvent A** | **% Solvent B** |
| --- | --- | --- |
| 0 | 98 | 2 |
| 15 | 98 | 2 |
| 50 | 55 | 45 |
| 60 | 55 | 45 |
| 62 | 5 | 95 |
| 75 | 5 | 95 |
| 75.5 | 98 | 2 |
| 80 | 98 | 2 |
