# Supplementary material for: Oral supplementation of gut microbial metabolite indole-3-acetate alleviates diet-induced steatosis and inflammation in mice
Source: eLife. 2024 Feb 27;12:RP87458. doi: 10.7554/eLife.87458 (PMC10942630; doi:10.7554/eLife.87458)
Supplement: Supplementary file 5. [file elife-87458-supp5.pdf]

# Proteomic Data processing workflow

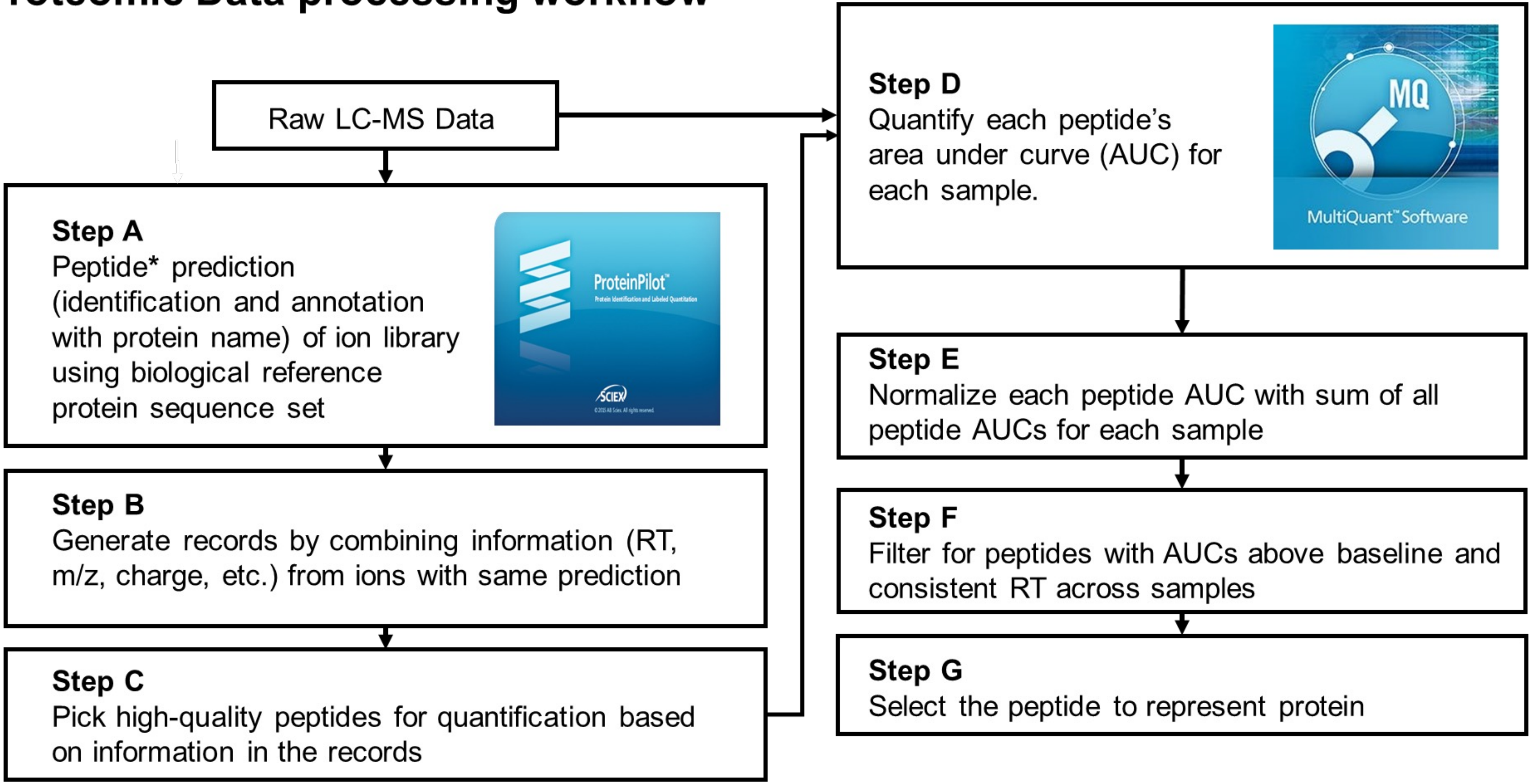


*Peptide refers to ion comprising an amino acid sequence with certain modification and charge, unless otherwise noted
