# Supplementary material for: Oral supplementation of gut microbial metabolite indole-3-acetate alleviates diet-induced steatosis and inflammation in mice
Source: eLife. 2024 Feb 27;12:RP87458. doi: 10.7554/eLife.87458 (PMC10942630; doi:10.7554/eLife.87458)
Supplement: Supplementary file 6. [file elife-87458-supp6.docx]

Supplementary File 6. LC-MS parameters for targeted proteomics

| **Protein** | **Selected Peptide** | **CE (V)** | **m/z** | **Charge** | **RT (min)** | **Product ion(s)** |
| --- | --- | --- | --- | --- | --- | --- |
| CD36 | VAIIESYK | 21.6 | 461.8 | 2 | 32.8 | 752.4 |
| Acox1 | TSNHAIVLAQLITR | 22.6 | 513 | 3 | 37.8 | 701.4 |
| Acaa1a | NTTPDELLSAVLTAVLQDVR | 32.5 | 719.1 | 3 | 53.3 | Sum of 901.5, 1014.6, 1113.7, 800.5, 729.4, 630.4, 517,3 |
| Acaa1b | DTTPDELLSAVLTAVLQDVK | 32.1 | 710.1 | 3 | 54.2 | 873.5 |
| Acads | IGIASQALGIAQASLDCAVK_Carbamidomethyl(C)@17 | 29.8 | 662.7 | 3 | 42.8 | 792.4 |
| Acadm | LLVEHQGVSFLLAEMAMK | 30.3 | 672.7 | 3 | 42.6 | 793.4 |
| Acadl | SPAHGISLFLVENGMK | 25.2 | 567.3 | 3 | 39 | 677.3 |
| Hadh | LLVPYLIEAVR | 30.5 | 643.4 | 2 | 43.1 | 960.6 |
| Acaa2 | TNVSGGAIALGHPLGGSGSR | 27 | 603.3 | 3 | 33.5 | 747.4 |
| Fasn | VLEALLPLK | 23.4 | 498.3 | 2 | 39.7 | 783.5 |
| Acab | VEANLLSSEESLSASELSGEQLQEHGDHSCLSYR_Carbamidomethyl(C)@30 | 45.1 | 941.2 | 4 | 38.1 | 1001.9 |
| Cat | GAGAFGYFEVTHDITR | 25.9 | 580.9 | 3 | 38.1 | 742.7 |
| Gpx1 | YVRPGGGFEPNFTLFEK | 29.4 | 653.3 | 3 | 39.5 | 498.3 |
| Hamp | DTNFPICIFCCK_Carbamidomethyl(C)@7; Carbamidomethyl(C)@10; Carbamidomethyl(C)@11 | 37.6 | 787.8 | 2 | 41.6 | 1097.5 |
| Tf | SAGWVIPIGLLFCK_Carbamidomethyl(C)@13 | 37.3 | 780.9 | 2 | 47 | 947.5 |

CD36: platelet glycoprotein 4; Acox1: peroxisomal acyl-coenzyme A oxidase 1; Acaa1a: peroxisomal 3-ketoacyl-CoA thiolase A; Acaa1b: peroxisomal 3-ketoacyl-CoA thiolase B; Acads: short-chain acyl-CoA dehydrogenase; Acadm: medium-chain acyl-CoA dehydrogenase; Acadl: long-chain acyl-CoA dehydrogenase; Hadh: hydroxyacyl-CoA dehydrogenase; Acaa2: mitochondrial 3-ketoacyl-CoA thiolase; Acab: acetyl-CoA carboxylase 2; Cat: catalase; Gpx1: Glutathione peroxidase 1; Hamp: hepcidin; Tf: serotransferrin
