# Supplementary material for: Oral supplementation of gut microbial metabolite indole-3-acetate alleviates diet-induced steatosis and inflammation in mice
Source: eLife. 2024 Feb 27;12:RP87458. doi: 10.7554/eLife.87458 (PMC10942630; doi:10.7554/eLife.87458)
Supplement: Supplementary file 7. [file elife-87458-supp7.docx]

Supplementary File 7. Primer sequences

| **Gene** | **Forward Primer Sequence** | **Reverse Primer Sequence** |
| --- | --- | --- |
| TNFα | TCTCATGCACCACCATCAAGGACT | TGACCACTCTCCCTTTGCAGAACT |
| IL-1β | TCCAGGATGAGGACATGAGCAC | GAACGTCACACACCAGCAGGTTA |
| β-actin | GGCTGTATTCCCCTCCATCG | CCAGTTGGTAACAATGCCATGT |
